# Diagnostic imaging in COVID-19 pneumonia: a literature review

**DOI:** 10.1007/s40477-021-00559-x

**Published:** 2021-02-15

**Authors:** Sarah Campagnano, Flavia Angelini, Giovanni Battista Fonsi, Simone Novelli, Francesco Maria Drudi

**Affiliations:** 1 Department of Radiological, Oncological and Path Sciences, Sapienza University of Rome, Rome, Italy; 2 Department of Surgery Pietro Valdoni, Sapienza University of Rome, Rome, Italy; 3 Department of Mechanical and Aerospace Engineering, Sapienza University of Rome, Rome, Italy

**Keywords:** COVID-19, SARS-CoV-2, US, Ultrasound, Pneumonia, CT

## Abstract

In December 2019 in Wuhan (China), a bat-origin coronavirus (2019-nCoV), also known as severe acute respiratory syndrome coronavirus 2 (SARS-CoV-2), was identified, and the World Health Organization named the related disease COVID-19. Its most severe manifestations are pneumonia, systemic and pulmonary thromboembolism, acute respiratory distress syndrome (ARDS), and respiratory failure. A swab test is considered the gold standard for the diagnosis of COVID-19 despite the high number of false negatives. Radiologists play a crucial role in the rapid identification and early diagnosis of pulmonary involvement. Lung ultrasound (LUS) and computed tomography (CT) have a high sensitivity in detecting pulmonary interstitial involvement. LUS is a low-cost and radiation-free method, which allows a bedside approach and needs disinfection of only a small contact area, so it could be particularly useful during triage and in intensive care units (ICUs). High-resolution computed tomography (HRCT) is particularly useful in evaluating disease progression or resolution, being able to identify even the smallest changes.

## Introduction

In December 2019 in Wuhan (China), a bat-origin coronavirus (2019-nCoV) was identified, also known as severe acute respiratory syndrome coronavirus 2 (SARS-CoV-2) [1], and the World Health Organization named the related disease COVID-19. COVID-19 is characterized by serious pathologies, such as pneumonia, necrotizing encephalopathy, systemic and pulmonary thromboembolism, acute respiratory distress syndrome (ARDS), respiratory failure, systemic inflammatory response, sepsis, and, rarely, gastro-intestinal and cutaneous involvement [2]. The main clinical presentation includes fever, dry cough, dyspnea, malaise, and/or non-specific upper respiratory tract infection symptoms. Some patients develop ARDS, requiring ventilatory support. The infection targets mainly the respiratory system, leading to interstitial pneumonia. The spike protein of the virus, also known as the S protein, binds to the angiotensin-converting enzyme 2 (ACE2) receptor expressed in the alveolar epithelium; this physiopathology explains the predominance of respiratory symptoms [3]. Histopathological studies in patients with COVID-19 showed inflammatory pulmonary changes characterized by alveolar edema, reactive alveolar epithelial hyperplasia, prominent proteinaceous exudates, and vascular congestion, as well as clusters with fibrinous material, multinucleated giant cells, and fibroblastic proliferation [4]. Laboratory findings of infected patients include lymphopenia, elevated C-reactive protein, and an elevated erythrocyte sedimentation rate. Genetic sequencing of SARS-CoV-2 has enabled the rapid development of point-of-care real-time reverse transcription-polymerase chain reaction (RT-PCR) diagnostic tests specific for COVID-19 [5, 6]. Identification of the viral pathogen through nucleic acid detection, usually from a swab test, is considered the gold standard for the diagnosis of COVID-19 [7], despite false negatives due to irregular sampling, laboratory error, insufficient viral material in the specimen, improper extraction of nucleic acid from clinical materials [7]. Radiologists play a crucial role in the rapid identification and early diagnosis of patients affected with COVID-19 pneumonia.

## Imaging features

Lung ultrasound (LUS) is an economic and easy tool, with a bedside approach, that can be used to diagnose COVID-19-related pulmonary involvement [8]. Computed tomography (CT) is the most sensitive technique for detecting early disease, assessing the nature and extent of lesions, and discovering minor changes that are often not visible on chest radiography [9]; it allows evaluating the disease’s evolution and the therapy outcome. Chest radiography of COVID-19 patients is not routinely recommended in clinical practice because it cannot detect COVID-19 in the early stage [7, 10]. Finally, 18F-fluorodeoxyglucose (FDG) positron emission tomography (PET)/CT cannot be routinely used in an emergency setting, and it is generally not recommended for infectious diseases, but it can be useful for differential diagnosis [11].

## Lung ultrasound

Ultrasound (US) can be used in the triage of symptomatic patients, in the assessment of the severity of lung damage, and in the assessment of the evolution of the disease [8]. It is a radiation-free method and can be safely used in children and pregnant women [8, 12, 13].

US needs disinfection of a small contact area and allows a bedside approach, thus preventing the dislocation of patients in the hospital, especially from the intensive care unit (ICU). The ability of US to assess pulmonary involvement is higher in severe cases, but it is reduced in mild or moderate cases [14, 15]. The disadvantages of US are the following: prolonged exposure of the examiner due to the difficulty of maintaining adequate distance from the patient, the use of uncomfortable personal protective equipment, specific disinfection of the transducers, no standards for reporting US changes, and inter-operator variability [16]. Furthermore, US examination is often performed in a COVID division (usually an ICU) with on-site available devices and not in a US laboratory with high-end machines [16].

For the detection of the interstitial syndrome in non-COVID patients, US has a sensitivity of 98% and a specificity of 88%, while chest radiography has a sensitivity of 60% and a specificity of 100% [17]. For consolidation, in non-COVID patients, US has a sensitivity of 93% and a specificity of 93%, compared with chest radiography that has a sensitivity of 68% and a specificity of 95% [17, 18].

US can also distinguish between cardiogenic and non-cardiogenic pulmonary edema and can exclude alternative causes of hypoxia in intensive care [19].

### Technique

During LUS scanning, the transducer is usually placed longitudinally, perpendicular to the ribs over the intercostal space. Different US protocols for studying the lung parenchyma are described in the literature. Some authors evaluate eight zones of the chest, four at each side (two anterior and two lateral), using low- and high-frequency probes [20]. Other authors [21] evaluate 14 areas (three posterior, two lateral, and two anterior) for 10 s, with an intercostal scan; for LUS examinations of patients who are not able to maintain a sitting position, the operator tries to have a partial view of the basal and dorsal regions.

**Fig. 1:**
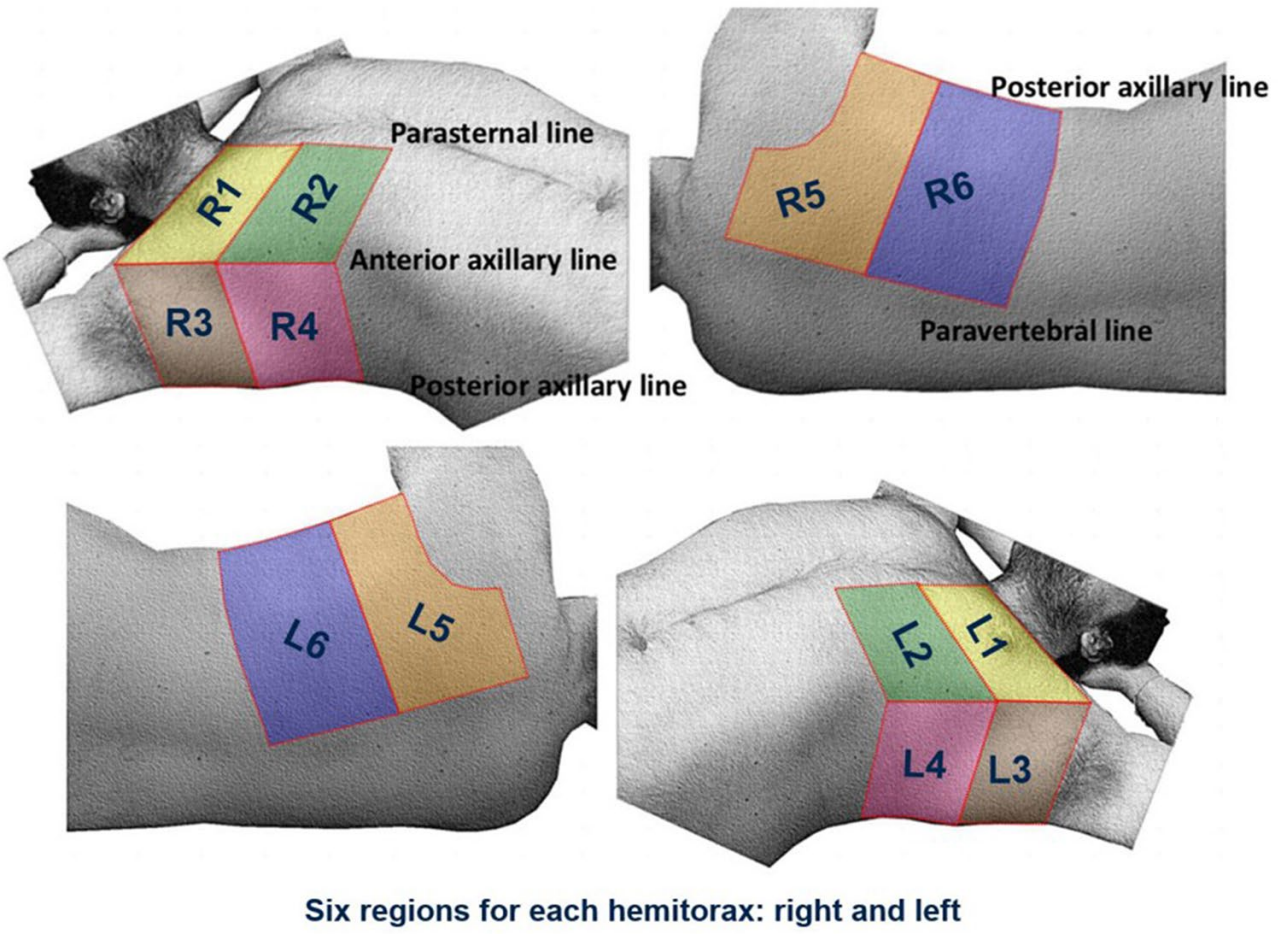
The 12-zone protocol in the evaluation of pulmonary parenchyma with US. Six zones on each hemithorax: anterior–superior (yellow), lateral-superior (beige), posterior-superior (orange), anterior-inferior (green), lateral-inferior (pink), and posterior-inferior (blue)

The most used protocol consists of the evaluation of three areas in each hemithorax (anterior, lateral, and posterior), using the anterior and posterior axillary lines as anatomical landmarks in supine patients. Each area is divided into two parts, superior and inferior. Therefore, six specific regions for each lung are scanned for 60 s [22, 23] (Fig. 1).

The BLUE protocol used by other authors [4] consists of the application of two hands side by side, without the thumbs, over the anterior chest with the wrists in the anterior axillary line and the upper little finger resting along the clavicle, defining three points: (1) the upper anterior point, corresponding to the base of the middle and ring fingers of the upper hand, which lies over the upper lobe; (2) the lower anterior point, corresponding to the middle of the palm of the lower hand (close to the nipple of a man), which lies over the middle or lingular lobe; and (3) the posterolateral point, which lies behind the posterior axillary line over the lower lobe [4].

Different probes are suitable for LUS [23]:

- Linear probes (with high superficial definition and low penetration capacity), which are useful in patients with a thin parietal wall, mainly in anterior fields, and in the evaluation of pleural pathologies.
- Phased-array and convex probes, which are more suitable for the examination of deep pathologies (consolidations and pleural effusions) and for thick parietal wall areas, mainly lateral and posterior.
- Microconvex probes, which are more flexible and are suitable for both superficial and deep pathology evaluation thanks to their wide frequency range [23].

**Fig. 2:**
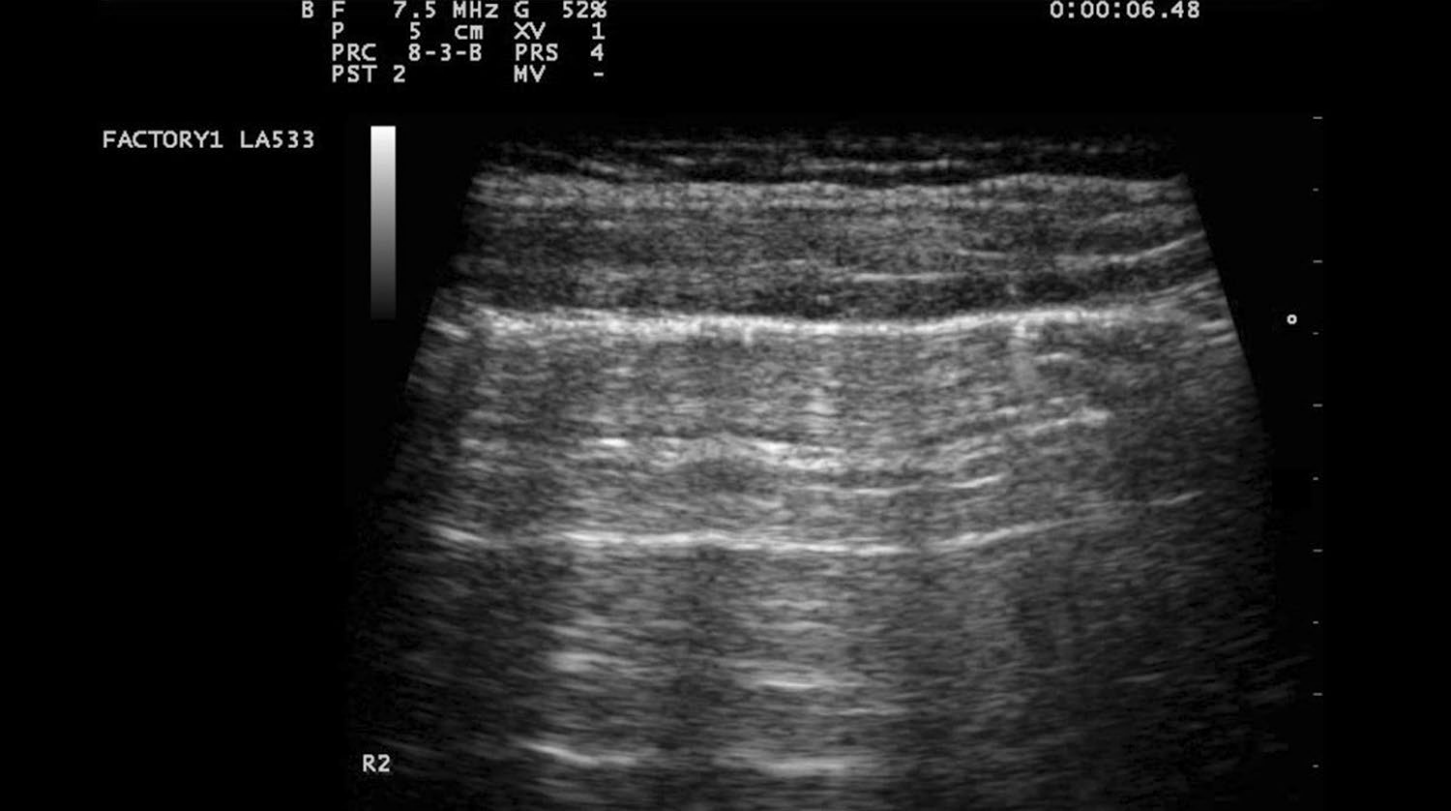
Normal lung US: A-lines are visible, indicating the presence of air below the pleural line

US signals are not transmitted through normally aerated lungs, and only the pleural line can be seen. It appears as a hyperechoic and sliding line, 2 mm thick, moving forward and backward with ventilation, resulting from the movement of the visceral pleura against the parietal pleura during the respiratory cycle [23]. The sliding line is an indicator of lung ventilation in the inspected area [4, 23]. Over the pleural line, horizontal reverberations are produced by the bouncing of the echo between the pleural line and the probe, the so-called A-lines [4] (Fig. 2). The extent of these artifacts varies depending on the ratio of air and fluid in the lung. The diagnostic findings of LUS imaging are based on the relative amounts of air and fluid present in the lung [4]. The increase in extra-vascular lung water produces vertical US artifacts resulting from the abnormal gas–tissue interface, the so-called B-lines, which appear as comet-tail vertical artifacts, arising from the pleural line, with different shapes and lengths [4]. They appear in US images when the lung loses normal aeration but is not completely consolidated [4]. They represent a reverberation artifact through edematous interlobular septa or alveoli [23]. Lung consolidation appears as a tissue-like echotexture (so-called hepatization). Within the consolidation, hyperechoic punctiform images can be seen, corresponding to air bronchograms [23]. A-lines and B-lines are determined by the degree of aeration, configuring different patterns [23]:

- A-lines beyond the pleural line: normal pulmonary aeration.
- Multiple and well-separated vertical B-lines: moderate decrease in lung aeration resulting from interstitial syndrome.
- Coalescent B-lines: more severe decrease in lung aeration resulting from partial filling of alveolar spaces.
- Lung consolidation: complete loss of aeration with persisting aeration of distal bronchioles (dynamic bronchograms) [23].
- Absence of A-lines and B-lines: a white lung.

The most used LUS score of aeration is the following. For each given region of interest, points are allocated according to the worst US pattern observed: normal = 0, well-separated B-lines = 1, coalescent B-lines = 2, and consolidation = 3 points [21–23].

### COVID-19 LUS findings

Although LUS is highly sensitive in detecting COVID‐19 pneumonia, there are no pathognomonic signs related to SARS‐CoV‐2 in the lungs. In fact, all the abnormal signs of COVID‐19 pneumonia are shared with all the interstitial and alveolar‐interstitial lung diseases, including viral pneumonia of different etiologies, *Pneumocystis jirovecii* pneumonia, idiopathic or secondary pulmonary fibrosis, hypersensitivity pneumonitis, congestive heart failure, and diffuse alveolar hemorrhage [24].

Although non-specific, B-lines are common in COVID-19. Peng et al. [25] first reported their appearance, which was later confirmed by other authors [26–29].

Characteristic findings of COVID-19 pulmonary involvement are the following: thickening of the pleural line and pleural line irregularity; B-lines in a variety of patterns (focal, multifocal, and confluent); and consolidations with occasional mobile air bronchograms; and, less frequently, pleural effusions [25–34]. The presence of pleural effusions may indicate that another diagnosis should be considered, such as bacterial pneumonia, bacterial superinfection, or congestive cardiac failure [33, 35]; the accuracy of US in detecting pleural effusions is 93% [33].

In the early stage of COVID-19, lung changes are more localized and are detected mainly in the subpleural regions of one or both lungs. Later, the pathology involves multiple lobes, leading to air loss and consolidations of some lesions surrounded by several B-lines [18]. In ARDS, including COVID-19-induced ARDS, US examination shows a white area in which neither A-lines nor separated B-lines are visible. This presentation is called a “white lung” [31].

The echostructure of the lung itself becomes visible with an air bronchogram, representing the air inside alveoli or bronchi surrounded by inflammation or pus; the pleural lines are so completely obscured [19, 31].

In their study, Huang et al. used a 3–17 MHz high-frequency linear array transducer and a 1–8 MHz convex array and scanned the thorax in 12 lung areas. LUS findings from 20 patients with confirmed COVID-19 were as follows: discontinuous or continuous/fused B-lines (37.9%), an unsmooth and rough pleural line (15%), multiple small patchy subpleural consolidations (22.1%), air bronchograms (15.4%), local pleural effusions around the lung lesions (18.8%), pleural thickening of 1–2 mm (14.6%), and poor blood flow in lesions (94.3%); both the right and the left posterior inferior lung were involved in 75% of cases [34].

Peng et al. studied 20 patients with COVID-19, dividing the thorax in 12 areas. Lung findings were the following: thickening and irregularity of the pleural line, B-lines in a variety of patterns (focal, multifocal, and confluent), consolidations in a variety of patterns (multifocal, non-translobar, and translobar), occasional mobile air bronchograms, appearance of A-lines during the recovery phase, and pleural effusions (uncommon) [25].

Lomoro et al. studied 58 patients in the emergency room and found a thickened pleural line (13.6% of cases), various patterns of B-lines (100%), consolidation (27.3%), pleural effusions (4.5%), and A-lines in (4.5%) [36].

In their study, Poggiali et al. analysed 12 patients and found thickening of the pleural line, an irregular pleural line, B-lines (focal and confluent), consolidations with air bronchograms, and pleural effusions (in a few cases) [37].

Yasukawa et al. analysed 10 patients using a bedside phased-array transducer while the patients were sitting up. US examinations were performed along the midclavicular line in the bilateral anterior chest wall and along the scapular line and in the interscapular regions of the posterior chest wall. The most frequent findings were the following: five or more B-lines (100%), a white lung (50%), three or four B-lines between two ribs (20%), and thick and irregular pleural lines (100%) [38].

Musolino et al. studied 10 patients with a wireless pocket device connected to a probe. They analysed 14 areas (three posterior, two lateral, and two anterior) per patient for 10 s and obtained the following results: vertical artifacts (70%), pleural irregularities (60%), areas of a white lung (10%), and subpleural consolidations (10%); no cases of pleural effusions were found [39].

Fonsi et al. [22] performed LUS examinations using the 12-zone method with the patients placed in both supine and lateral positions, using convex and linear vascular transducers (2.5–5 and 7.5–12 MHz, respectively). Among the 44 patients with COVID-19, they found thickened pleural lines (86% of cases), B-lines (100%; in different patterns), consolidations (45%), bilateral distributions (75%), and pleural effusions (18%).

**Fig. 3:**
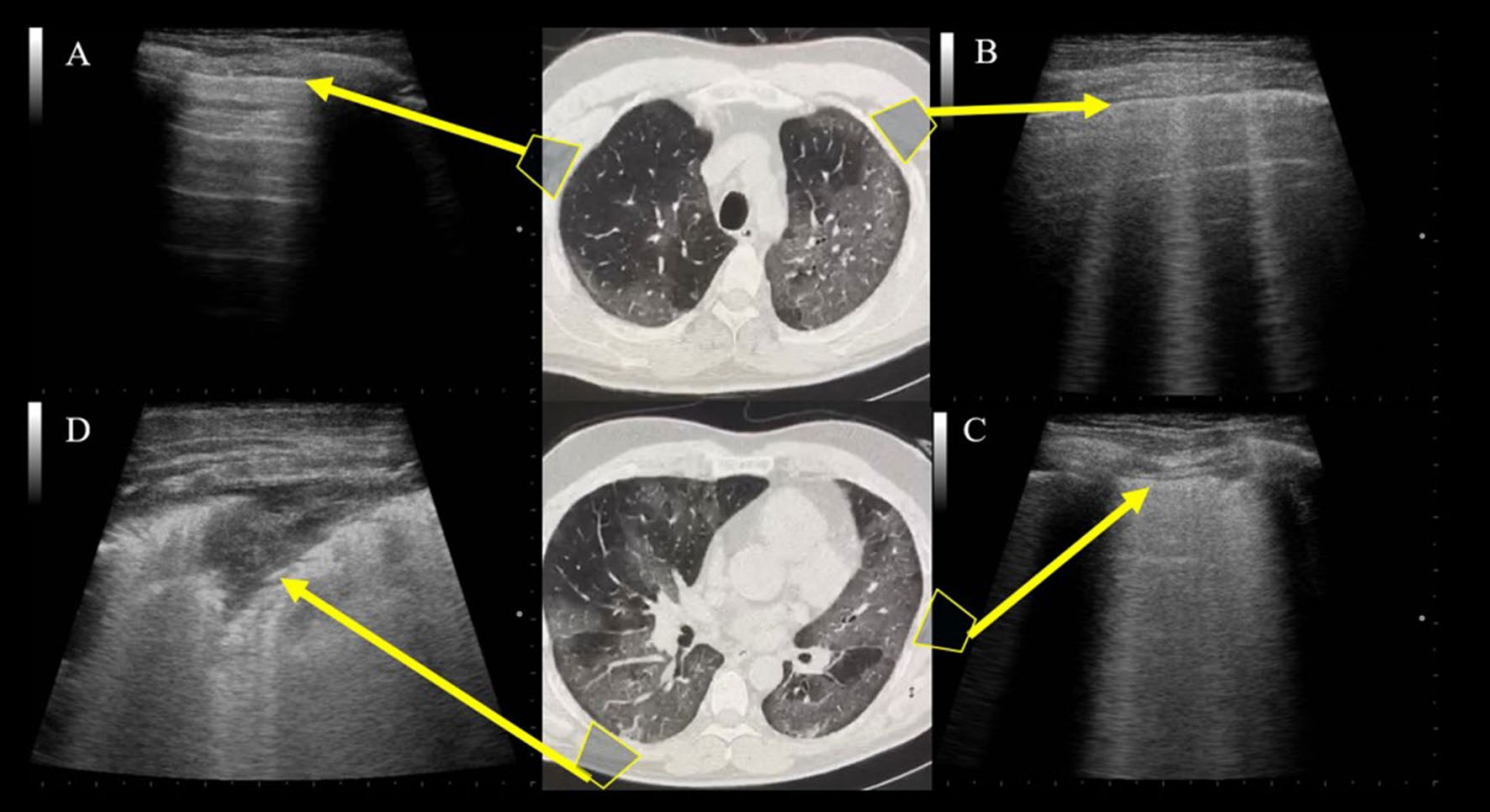
Imaging correlation between LUS and chest CT scans of the same patients. **a** A-lines in normally aerated parenchyma. **b** Well-separated B-lines, corresponding to subpleural GGOs. **c** Coalescent B-lines, corresponding to more severe GGOs resulting from partial filling of alveolar spaces. **d** Lung consolidation

All these US findings are summarized in Table 1. All the patients who underwent LUS were in an emergency department with swab-confirmed COVID-19 infection. Some authors evaluated the relationship between LUS signs and CT findings. Lopes et al. [24] found that patients with more than two B‐lines on LUS had more ground-glass opacity (GGO) areas on CT than those with fewer than two B‐lines on LUS. Patients with subpleural consolidations on LUS had more areas of consolidation on CT than those without subpleural consolidations on LUS, and patients with higher LUS aeration scores had more extensive and more advanced disease on CT. Similar results were obtained by Peng et al. [25], Lomoro et al. [36], and Poggiali et al. [37] (Fig. 3). Peng et al. and Huang et al. have demonstrated a high correlation between LUS and chest CT scans in the same patients [25, 34].

**Table 1:** The most frequent chest US findings reported in mentioned studies and their relative frequency

Author	N. of Patient	Type of Patient	B-Line	PleuralLine irreg	White lung	Consolidation	Pleural effusion	Broncho-grams	Pleuralthickening	A-lines
Huang et al. [34]	20	ED**	37.9%	15%	–	22.1%	18.8%	15.4%	14.%	–
Peng et al. [25]	20	–	NA*	NA*	–	NA*	NA*	NA*	NA*	NA*
Lomoro et al. [36]	58	ED**	100%	–	–	27.3%	4.5%	–	13.6%	4.5%
Poggiali et al. [37]	12	ED**	NA*	NA*	–	NA*	NA*	NA*	NA*	–
Yusukawa et al. [38]	10	ED**	100%	100%	50%	–	–	–	100%	–
Musolino et al. [39]	10	ED**	70%	60%	10%	10%	0%	–	–	–
Fonsi et al. [22]	44	ED**	100%	–	–	45%	0%	39%	86%	20%

*We entered NA (not available) in those cases where the authors didn’t provide a more accurate value for the findings

**We entered ED (Emergency Department) in those cases where patients have been subjected to LUS in the emergency Department

## Computed tomography

CT is more sensitive and specific than X-ray spectroscopy and can identify lung interstitial involvement in the early stages of COVID-19. It is often used in patients with a high clinical suspicion of COVID-19 in the screening and preliminary diagnosis of pneumonia [40, 41], although a normal chest CT scan does not exclude COVID-19 [2].

The typical high-resolution computed tomography (HRCT) pattern consists of single or multiple GGOs, with mainly a subpleural distribution, crazy paving, and patchy pattern with segmental pulmonary consolidations [42, 43].

COVID-19 has different manifestations at different stages of the disease. Most patients have single or multiple GGOs in the early stages of the disease, which continue to expand with disease progression. The reported prevalence of GGO varies between 46 and 100%, and GGO is usually found in the early phases of the disease and/or in mild pulmonary infection [44–67] (Table 2). In the later stages of COVID-19, GGO is often combined with other imaging features, such as pulmonary consolidation, the appearance of crazy paving, and pleural effusion [50, 68–72].

**Table 2:** The most frequent chest CT findings reported in mentioned studies and their relative frequency

Author	N. of patient	GGO	Consolidation	GGO + Consolidation	Crazy paving	Spider web sing	Broncho-grams	Pleural effusion	Reversed halo sign	Air bubble
Ai et al. [60]	888	46%	50%	–	–	–	–	–	–	–
Bai et al. [63]	219	91%	69%	64%	5%	–	14%	15%	5%	–
Bernheim et al. [47]	121	34%	2%	41%	5%	–	–	0.8%	1.7%	–
Cheng et al. [64]	11	100%	54.4%	63.6%	–	–	72.7%	0%	–	9.1%
Chung et al. [46]	21	57%	0%	29%	19%	–	–	0%	–	–
Fan et al [67]	150	83%	–	–	35%	–	36%	4%	–	
Fang et al. [45]	51	72%	–	–	–	–	–	–	–	–
Han et al [53]	108	60%	6%	41%	40%	–	48%	0%	–	–
Li et al [61]	83	97.6%	63.9%	–	36.1%	25.3%	–	8.4%	–	–
Li et al [58]	51	90.2%	5.9%	54.9%	70.6%	–	68.6%	2%	3.9%	–
Ng et al [51]	21	86%	62%	–	–	–	–	0%	9.6%	–
Pan et al [52]	21	71%	91%	–	81%	–	–	–	–	–
Pan et al [50]	63	85.7%	19%	–	–	–	–	–	–	–
Shi et al [69]	34	65%	17%	–	10%	–	47%	5%	–	10%
Song et al. [49]	51	77%	55%	59%	75%	–	80%	8%	–	–
Wang et al. [66]	35	74.2%	60.2%	–	36.6%	–	–	8.6%	15.1%	12.9%
Wu et al. [48]	80	91%	63%	–	29%	25%	–	6%	–	–
Xie et al [44]	5	100%	–	40%	–	–	–	–	–	–
Xiong et al. [62]	42	100%	55%	–	–	–	33%	12%	–	–
Xu et al [57]	90	72%	13%	–	12%	–	8%	4%	–	–
Xu et al. [71]	41	73%	37%	61%	80%	–	54%	7.1%	–	–
Yang et al. [7]	149	–	–	–	–	–	54.4%	6.7%	–	8.1%
Zhao et al. [55]	101	86.1%	43.6%	64.4%	–	–	–	–	–	–
Zhou et al. [56]	62	40.3%	33.9%	–	62.9%	–	72.6%	9.7%	–	54.8%

Pulmonary consolidation in patients with COVID-19 is regarded as a sign of disease progression. Pan et al. found that pulmonary consolidation is rare in the early stages of COVID-19, but with the progression of the disease, it gradually appears. In the later stages of COVID-19, the range of pulmonary consolidation becomes larger and diffuse [49, 52, 68–72]. The reported prevalence of a crazy-paving pattern in COVID-19 patients varies between 5 and 89% [44–67, 73].

The reported prevalence of thromboembolic events in patients with COVID-19 is higher than that in the healthy population. Therefore, when CT shows peripherally located, triangular-shaped consolidation areas, pulmonary thromboembolism and infarction should be considered in the differential diagnosis [74, 75]. Grillet et al. reported a 23% prevalence of pulmonary embolism in COVID-19 patients with severe clinical features [74]. Leonard-Lorant et al. reported that 22 of 106 patients (30%) with COVID-19 had acute pulmonary thromboembolism [76].

Bai et al. [63] compared the CT findings in patients with COVID-19 pneumonia with those in patients with non-COVID-19 pneumonia, and they found that pulmonary vascular enlargement (PVE) was significantly associated with COVID-19. Moreover, PVE was reported in 45.2–89.2% of COVID-19 patients and should be attributed to vascular wall inflammatory infiltration [55, 56, 58, 63, 66].

Pleural pathologies, such as pleural effusion and focal pleural thickening, have rarely been reported in cases with COVID-19, and pleural pathologies are usually seen in the later stages of the disease [52, 62]. The prevalence of pleural effusion in COVID-19 patients has been reported to range from 0 to 20% [44–67, 73] (Table 2).

The air bubble sign is a small air-containing space that may be related to the pathological expansion of alveolar sacs or bronchioles or the absorption process of consolidation; the reported prevalence of air bubble signs in patients with COVID-19 pneumonia ranges from 8.1 to 54.8% [54, 57, 62–66, 77].

The reversed halo sign (RHS) indicates a central GGO surrounded by denser ring-like (crescentic shape) consolidation, also known as the Atoll sign [78], and it has been reported in several COVID-19 cases.

The spider web sign was defined by Wu et al. [48]; they described it as an angular- or triangular-shaped peripheral GGO with interstitial thickening, like a spider web in a corner. Li et al. [61] detected the spider web sign in 21 out of 83 (25.3%) patients with COVID-19 pneumonia.

Lymphadenopathies have been reported in 0%–29% of patients with COVID-19 [48, 49, 54, 61].

Pericardial effusion has rarely been reported in COVID-19 patients, which may indicate the occurrence of myocardial and/or pericardial inflammation. Li et al. [61] have reported that COVID-19 patients with severe and critical diseases showed a higher frequency of pericardial effusion than non-critical patients. Recently, Xu et al. [54] found pericardial effusion in one out of 90 (1.1%) patients with COVID-19 pneumonia.

An expert consensus statement by the Radiological Society of North America (RSNA) suggested standardized reporting and classification of imaging features for COVID-19 pneumonia [79].

Salehi et al. have defined a new classification by analysing 37 published studies that examined the diagnostic chest CT findings of COVID-19 patients. The classification contains five categories [80]:

- COVID-RADS 0: normal CT findings,
- COVID-RADS 1: atypical CT findings (inconsistent with COVID-19),
- COVID-RADS 2A: fairly typical findings,
- COVID-RADS 2B: a combination of atypical findings and typical/fairly typical findings, and
- COVID-RADS 3: typical CT findings.

Recently, the Dutch Radiological Society has defined a new classification (CO-RADS) for pulmonary involvement in cases presenting with moderate and severe symptoms of COVID-19. In this standardized assessment (CO-RADS), a substantial agreement was found among eight observers (Fleiss’ kappa of 0.47, 95% CI [0.45, 0.49]), and the discriminatory power of CO-RADS for diagnosing COVID-19 was high (with an area under the curve of 0.91, 95% CI [0.85, 0.97]) [81].

For the staging of lung involvement, Chung et al. [46] and Li et al. [82] used the same scoring, as follows: none (0%) = score 0, minimal (1%–25%) = score 1, mild (26%–50%) = score 2, moderate (51–75%) = score 3, and severe (76–100%) = score 4. An overall lung involvement score was reached by summing the five lobe scores (0–20).

## Chest radiographs

Although a chest X-ray is less sensitive than a CT scan, it may be used as a first-line approach because of its availability and easiness of decontamination. It is useful in cases in which the patient cannot be moved to the CT scanner in a radiology department [83]. A chest radiograph has low sensitivity in the first days of COVID-19; in fact, it may be normal early in the clinical course and tends to peak 10–12 days after the onset of clinical symptoms [83–85]. GGOs may not be thick enough to be seen on radiographs, and if they have a basal and retro-cardiac location, it may be difficult to see them because they are obscured by the overlying diaphragm in the frontal view and by mediastinal structures in the lateral view [86]. As the disease progresses, chest radiographs can detect multiple patchy opacities that become confluent and, in severe cases, may appear as a white lung. In more advanced cases, in addition to GGO and consolidation, even pleural fluid has been reported [1, 7, 36, 85–92].

In their study, Wong et al. [85] analysed the chest radiographs of 64 patients affected by COVID-19 infection, confirmed by RT-PCR on nasopharyngeal swabs and throat swabs, revealing that 51 out of 64 patients had abnormalities on their chest X-rays (CXRs) during illness. On baseline CXR, consolidation was the most common finding (47%), followed by GGO (33%). Peripheral (41%) and lower zone distribution (50%) were the more common locations, and most patients had bilateral involvement (50%). Pleural effusion was found in two cases (3%). Out of 58 (91%) patients who tested positive on the initial RT-PCR, 38 (59%) had abnormalities on the first CXR. Six patients (9%) were negative on the initial RT-PCR but demonstrated abnormalities on the first CXR. Of these six patients, five subsequently tested positive after 24 h, and one tested positive after 48 h. The detection rate of the first RT-PCR was 58/64, a 91% sensitivity (95% CI [83, 96]), which was higher than that of the first CXR (44/64, a 69% sensitivity, 95% CI [56, 80]) (*p* = 0.009).

In their study, Pakray et al. [93] performed imaging on 227 patients with either a positive RT-PCR or a high clinical suspicion of COVID-19 infection. Out of the total 227 patients imaged, 174 (76.6%) were RT-PCR positive, 19 (8.3%) were RT-PCR negative, and 34 (15%) were not tested or pending. Of the 173 RT-PCR positive patients, 155 had abnormal imaging (89.6%), including 86% of the CXRs and 100% of the CTs. Among the abnormal findings, the most common manifestations were mixed airspace and interstitial opacities (74, 39.8%), multifocality (99, 53.2%), and bilaterality (165, 88.7%).

In their study with 99 patients, Chen et al. [89] found that bilateral pneumonias were the most common findings on chest radiographs.

Ming-Yen et al. [94] performed CXR examinations on five patients; two patients showed normal CXR findings, with CT examinations performed on the same day showing GGOs. The other three CXR examinations showed consolidation. One CXR examination showed lower zone predominance, while the other two CXR examinations did not show any zonal predominance. In these three patients, the CXR examinations did not demonstrate the peripheral predominance that was visible on their respective CT examinations.

Lomoro et al. [36] found a CXR sensitivity of 84% (27 out of 32). Of 170 non-hospitalized patients with mild symptoms, Bandirali et al. [94] found 100 (58.8%) abnormal CXRs suggestive of COVID-19; however, RT-PCR confirmation was not performed.

All these chest radiograph findings are summarized in Table 3.

**Table 3:** The most frequent chest radiograph findings reported in mentioned studies and their relative frequency

Author	N. of patient	Abnormal Chest X-ray	Consolidation	GGO	Distribution	Pleural effusion	Bilateral	Sensitivity
Wong et al. [85]	64	80%	47%	33%	Peripheral/lower zone 50%	3%	50%	69%
Pakray et al. [93]	173	86%	39.8%	–	Multifocality 99.53%	–	88.7%	–
Chen et al. [89]	99			14%			76%	
NG Ming-Yen et al. [94]	5	60%	60%	–	Lower zone 20%	–	–	–
Lomoro et al. [36]	32	84%	–	–	-	–	100%	84%
Bandirali et al. [94]	170	58.8%	–	–	-	–	–	–
Yoon et al. [90]	9	56%	NA*	NA*	Lower zone 50%	–	–	33%

*We entered NA (not available) in those cases where the authors didn’t provide a more accurate value for the findings.

## MRI

Although it is not relevant for the evaluation of lung disease, magnetic resonance imaging (MRI) can contribute to the diagnostic pathway of patients with symptoms from the central nervous system, such as acute stroke, skeletal muscle injuries, impaired consciousness, or acute necrotizing hemorrhagic encephalopathy.

MRI can have a role in the diagnosis of COVID-19 complications, such as cardiac complications or persistent myositis [95, 96]. MRI can also demonstrate incidental findings related to COVID-19 in the pulmonary parenchyma. The pulmonary distribution of COVID-19 on MRI is consistent with CT and CXR, including basilar and peripheral predominant disease. On MRI, parenchymal changes of COVID-19 pneumonia appear as regions of abnormally increased signal intensity on both T1- and T2-weighted sequences, corresponding to the GGOs or consolidative opacities seen on CXR and CT [97].

## FDG-PET

FDG-PET is not used in an emergency, but it is useful in identifying inflammatory processes in the lungs, in monitoring disease progression, and in following treatment [97].

During viral infection, the host response triggers a rapid surge in inflammatory mediators, including neutrophils, monocytes, and chemokines. During acute infection, the neutrophils depend on anaerobic glycolysis to maintain cellular activity, and this increase in glucose requirement causes an increase in FDG uptake on PET/CT [98].

Lung lesions in COVID-19 pneumonia have high 18F-FDG uptake [11, 99, 100]. In their study, Chunxia et al. [11] used 18F-FDG PET/CT on four patients admitted to the hospital with respiratory symptoms and fever when the COVID-19 outbreak was still unrecognized and virus infectivity was unknown. All patients had peripheral GGOs and/or lung consolidations in more than two pulmonary lobes, characterized by a high 18F-FDG uptake (with a maximum standardized uptake value [SUV] range of 2.2–4.6) with lymph node involvement. Reported SUVs ranged from 4.6 to 12.2 [99, 100]. Tumors presenting as GGOs are unlikely to be FDG-avid [11].

PET/CT also has the potential as a whole-body non-invasive examination to assess chronic end-organ complications.

## Conclusion

In COVID-19, imaging has an important role in the diagnostic steps, as swabs can sometimes be negative. Chest X-ray has low sensitivity, especially in the early phase of the disease and in mild cases. In contrast, LUS and HRCT have a high sensitivity in detecting pulmonary interstitial involvement. The greater sensitivity of LUS compared with CT can be explained by the fact that SARS‐CoV‐2 often induces lesions in the posterior and inferior areas of the lung, in the subpleural region, which is particularly suitable for LUS investigations. Moreover, COVID‐19 pneumonia is characterized by alveolar‐interstitial damage with inflammatory exudation/edema, and LUS is highly sensitive to variations in the balance between air and fluids in the lung.

LUS is a low-cost and radiation-free method, useful in children and pregnant women. It allows a bedside approach and needs disinfection of only a small contact area, so it could be particularly useful during triage and in ICUs. Moreover, LUS might even be performed in patients’ homes, reducing the waiting times for CT in emergency departments, which are often overcrowded.

The main disadvantages of LUS are the difficulty of maintaining distance from the patient and the inter-operator variability. HRCT is particularly useful in evaluating disease progression or resolution, being able to objectively identify even the smallest changes.

## References

[1] Zhu N, Zhang D, Wang W, Li X, Yang B (2020). A novel coronavirus from patients with pneumonia in China, 2019. N Engl J Med.

[2] Furkan U, Recep S (2020). Chest CT features of the novel coronavirus disease (COVID-19). Turk J Med Sci.

[3] Zhou P, Yang X-L, Wang X-G, Ben Hu, Zhang L, Zhang W, Si H-R, Zhu Y, Li B, Huang C-L, Chen H-D, Chen J, Luo Y, Guo H, Jiang R-D, Liu M-Q, Chen Y, Shen X-R, Wang Xi, Zheng X-S, Zhao K, Chen Q-J, Deng F, Liu L-L, Yan B, Zhan F-X, Wang Y-Y, Xiao G-F, Shi Z-L (2020). A pneumonia outbreak associated with a new coronavirus of probable bat origin. Nature.

[4] Sultan LR, Sehgal CM (2020). Review of early experience in lung ultrasound (LUS) in the diagnosis and management of COVID-19. Ultrasound Med Biol..

[5] Wang C, Horby PW, Hayden FG, Gao GF (2020). A novel corona-virus outbreak of global health concern. Lancet.

[6] Yu F, Du L, Ojcius DM, Pan C, Jiang S (2020). Measures for diagnosing and treating infections by a novel coronavirus responsible for a pneumonia outbreak originating in Wuhan, China. Microbes Infect.

[7] Yang W, Sirajuddin A, Zhang X, Liu G, Teng Z, Zhao S, Lu M (2020). The role of imaging in 2019 novel coronavirus pneumonia (COVID-19). Eur Radiol.

[8] Dudea SM (2020). Ultrasonography and SARS-CoV 2 infection: a review of what we know and do not yet know. Med Ultrason.

[9] Jin YH, Cai L, Cheng ZS, Cheng H, Deng T, Fan YP, Fang C, Huang D, Huang LQ, Huang Q, Han Y, for the Zhongnan Hospital of Wuhan University Novel Coronavirus Management and Research Team, Evidence-Based Medicine Chapter of China International Exchange and Promotive Association for Medical and Health Care (CPAM) (2020). A rapid advice guideline for the diagnosis and treatment of 2019 novel coronavirus (2019-nCoV) infected pneumonia (standard version). Mil Med Res..

[10] Rubin GD, Ryerson CJ, Haramati LB, Sverzellati N, Kanne JP, Raoof S, Schluger NW, Volpi A, Yim JJ, Martin IB, Anderson DJ (2020). The role of chest imaging in patient management during the COVID-19 pandemic: a multinational consensus statement from the fleischner society. Radiology.

[11] Qin C, Liu F, Yen T-C, Lan X (2020). 18F-FDG PET/CT findings of COVID-19: a series of four highly suspected cases. Eur J Nucl Med Mol Imaging.

[12] Vitale V, Rossi E, Di Serafino M, Minelli R, Acampora C, Iacobellis F, D’Errico C, Esposito A, Esposito F, Vallone G, Zeccolini M (2018). Pediatric Encephalic ultrasonography: the essentials. J Ultrasound.

[13] Minella R, Minelli R, Rossi E, Cremone G, Tozzi A (2020). Gastroesophageal and gastric ultrasound in children: the state of the art. J Ultrasound.

[14] Lu W, Zhang S, Chen B, Chen J, Xian J, Lin Y, Shan H, Su ZZ (2020). A Clinical Study of Noninvasive Assessment of Lung Lesions in Patients with Coronavirus Disease-19 (COVID-19) by Bedside Ultrasound. Ultraschall Med.

[15] Guarracino F, Vetrugno L, Forfori F, Corradi F, Orso D, Bertini P, Ortalda A, Federici N, Copetti R, Bove T (2020). Lung, heart, vascular, and diaphragm ultrasound examination of COVID-19 patients: a comprehensive approach. J Cardiothorac Vasc Anesth.

[16] Piscaglia F, Stefanini F, Cantisani V, Sidhu PS, Barr R, Berzigotti A, Chammas MC, Correas JM, Dietrich CF, Feinstein S, Huang P (2020). Benefits, open questions and challenges of the use of Ultrasound in the COVID-19 pandemic era. The views of a panel of world- wide international experts. Ultraschall Med.

[17] Sekiguchi H, Schenck LA, Horie R, Suzuki J, Lee EH, McMenomy BP, Chen TE, Lekah A, Mankad SV, Gajic O (2015). Critical care ultrasonography differentiates ARDS, pulmonary edema, and other causes in the early course of acute hypoxemic respiratory failure. Chest.

[18] Zanforlin A, Tursi F, Marchetti G, Pellegrino G, Vigo B, Smargiassi A, Inchingolo R, Centanni S, Gasparini S, Blasi F, Soldati G, Papa GFS (2018). Clinical use and barriers of thoracic ultrasound: a survey of italian pulmonologists. Eur Respirat J.

[19] Boccatonda A, Decorato V, Cocco G, Marinari S, Schiavone C (2018). Ultrasound evaluation of diaphragmatic mobility in patients with idiopathic lung brosis: a pilot study. Multidiscip Respir Med.

[20] Di Serafino M, Notaro M, Rea G, Iacobellis F, Delli Paoli V, Acampora C, Ianniello S, Brunese L, Romano L, Vallone G (2020). The lung ultrasound: facts or artifacts? In the era of COVID-19 outbreak. Radiol Med.

[21] Soldati G, Smargiassi A, Inchingolo R, Buonsenso D, Perrone T, Briganti DF, Perlini S, Torri E, Mariani A, Mossolani EE, Tursi F (2020). Proposal for international standardization of the use of lung ultrasound for patients with COVID-19 a simple, quantitative reproducible method. J Ultrasound Med..

[22] Fonsi GB, Sapienza P, Brachini G, Andreoli C, De Cicco ML, Cirillo B, Meneghini S, Pugliese F, Crocetti D, Fiori E, Mingoli A (2020). Is lung ultrasound imaging a worthwhile procedure for severe acute respiratory syndrome coronavirus 2 pneumonia detection?. J Ultrasound Med..

[23] Bouhemad B, Mongodi S, Via G, Rouquette I (2015). Ultrasound for “Lung Monitoring” of ventilated patients. Anesthesiology.

[24] Lopes AJ, Mafort TT, da Costa CH, Rufino R, de Cássia FM, Kirk KM, Cobo CG, da Costa HD, da Cruz CM, Mogami R (2020). Comparison between lung ultrasound and computed tomographic findings in patients with COVID-19 pneumonia. JUM.

[25] Peng QY, Wang XT, Zhang LN (2020). Findings of lung ultrasonography of novel corona virus pneumonia during the 2019–2020 epidemic. Intensive Care Med.

[26] Buonsenso D, Piano A, Raffaelli F, Bonadia N, de Gaetano DK, Franceschi F (2020). Point-of-care lung ultrasound findings in novel coronavirus disease-19 pnemoniae: a case report and potential applications during COVID-19 outbreak. Eur Rev Med Pharmacol Sci.

[27] Thomas A, Haljan G, Mitra A (2020). Lung ultrasound findings in a 64-year-old woman with COVID-19. CMAJ.

[28] Vetrugno L, Bove T, Orso D, Barbariol F, Bassi F, Boero E, Ferrari G, Kong R (2020). Our Italian experience using lung ultrasound for identification, grading and serial follow-up of severity of lung involvement for management of patients with COVID-19. Echocardiography.

[29] Kalafat E, Yaprak E, Cinar G, Varli B, Ozisik S, Uzun C, Azap A, Koc A (2020). Lung ultrasound and computed tomographic findings in pregnant woman with COVID-19. Ultrasound Obstet Gynecol.

[30] Lichtenstein D, Goldstein I, Mourgeon E, Cluzel P, Grenier P, Rouby JJ (2004). Comparative diagnostic performances of auscultation, chest radiography, and lung ultrasonography in acute respiratory distress syndrome. Anesthesiology.

[31] Miller A (2016). Practical approach to lung ultrasound. BJA Educ.

[32] Koegelenberg CFN, Von Groote-Bidlingmaier F, Bolliger CT (2012). Transthoracic ultrasonography for the respiratory physician. Respiration.

[33] Kulkarni S, Down B, Jha S (2020). Point-of-care lung ultrasound in intensive care during the COVID-19 pandemic. Clin Radiol.

[34] Huang Y, Wang S, Liu Y, Zhang Y, Zheng C, Zheng Y, Zhang C, Min W, Zhou H, Yu M, Hu M (2020). A preliminary study on the ultrasonic manifestations of peripulmonary lesions of non-critical novel coronavirus pneumonia (COVID-19). SSRN Electron J.

[35] Tufano A, Minelli R, Di Lascio G, Delicato G, Baffigo G, Signore S (2020). Infected kidney stone progressing to perinephric abscess and thoracic empyema. Archivio Italiano Di Urologia E Andrologia.

[36] Lomoro P, Verde F, Zerboni F, Simonetti I, Borghi C, Fachinetti C, Natalizi A, Martegani A (2020). COVID-19 pneumonia manifestations at the admission on chest ultrasound, radiographs, and CT: single-center study and comprehensive radiologic literature review. Eur J Radiol Open Elsevier.

[37] Poggiali E, Dacrema A, Bastoni D, Tinelli V, Demichele E, Mateo Ramos P, Marcianò T, Silva M, Vercelli A, Magnacavallo A (2020). Can Lung US Help Critical Care Clinicians in the Early Diagnosis of Novel Coronavirus (COVID-19) Pneumonia?. Radiology.

[38] Yasukawa K, Minami T (2020). Point-of-care lung ultrasound findings in patients with novel coronavirus disease (COVID-19) pneumonia. Am J Trop Med Hyg.

[39] Musolino AM, Supino MC, Buonsenso D, Ferro V, Valentini P, Magistrelli A, Lombardi MH, Romani L, D’Argenio P, Campana A (2020). Lung ultrasound in children with COVID-19: preliminary findings. Ultrasound Med Biol.

[40] Memish ZA, Al-Tawfiq JA, Assiri A (2014). Middle east respiratory syndrome coronavirus disease in children. Pediatr Infect Dis J.

[41] Rao TA, Paul N, Chung T, Mazzulli T, Walmsley S, Boylan CE, Provost Y, Herman SJ, Weisbrod GL, Roberts HC (2003). Value of CT in assessing probable severe acute respiratory syndrome. Am J Roentgenol..

[42] Kanne JP (2020). Chest CT findings in 2019 novel coronavirus (2019-nCoV) infections from Wuhan, China: key points for the radiologist. Radiology.

[43] Kim H (2020). Outbreak of novel coronavirus (COVID-19): what is the role of radiologists?. Eur Radiol.

[44] Xie X, Zhong Z, Zhao W, Zheng C, Wang F, Liu J (2020). Chest CT for typical 2019-nCoV pneumonia: relationship to negative RT-PCR testing. Radiology.

[45] Fang Y, Zhang H, Xie J, Lin M, Ying L, Pang P, Ji W (2020). Sensitivity of chest CT for COVID-19: comparison to RT-PCR. Radiology.

[46] Chung M, Bernheim A, Mei X, Zhang N, Huang M, Zeng X, Cui J, Wenjian Xu, Yang Y, Fayad ZA, Jacobi A, Li K, Li S, Shan H (2020). CT imaging features of 2019 novel coronavirus (2019-nCoV). Radiology.

[47] Bernheim A, Mei X, Huang M, Yang Y, Fayad ZA, Zhang N, Diao K, Lin B, Zhu X, Li K, Li S, Shan H, Jacobi A, Chung M (2020). Chest CT ndings in coronavirus disease-19 (COVID-19): relationship to duration of infection. Radiology.

[48] Jiong Wu, Xiaojia Wu, Zeng W, Guo D, Fang Z, Chen L, Huang H, Li C (2020). Chest CT findings in patients with corona virus disease 2019 and its relationship with clinical features. Invest Radiol.

[49] Song F, Shi N, Shan F, Zhang Z, Shen J, Hongzhou Lu, Ling Y, Jiang Y, Shi Y (2020). Emerging 2019 novel coronavirus (2019-nCoV) pneumonia. Radiology.

[50] Pan Y, Guan H, Zhou S (2020). Initial CT findings and temporal changes in patients with the novel coronavirus pneumonia (2019-nCoV): a study of 63 patients in Wuhan, China. Eur Radiol.

[51] Ng MY, Lee EY, Yang J, Yang F, Li X, Wang H, Lui MM, Lo CS, Leung B, Khong PL, Hui CK (2020). Imaging profile of the COVID-19 infection: radiologic findings and literature review. Radiol Cardiothorac Imaging.

[52] Pan F, Ye T, Sun P, Gui S, Liang Bo, Li L, Zheng D, Wang J, Hesketh RL, Yang L, Zheng C (2019). Time course of lung changes on chest CT during recovery from 2019 novel coronavirus (COVID-19) pneumonia. Radiology.

[53] Rui Han Lu, Huang HJ, Dong J, Peng H, Zhang D (2020). Early clinical and CT manifestations of coronavirus disease 2019 (COVID-19) pneumonia. Am J Roentgenol.

[54] Xi Xu, Chengcheng Yu, Jing Qu, Zhang L, Jiang S, Huang D, Chen B, Zhang Z, Guan W, Ling Z, Jiang R, Tianli Hu, Ding Y, Lin L, Gan Q, Luo L, Tang X, Liu J (2020). Imaging and clinical features of patients with 2019 novel coronavirus SARS-CoV-2. Eur J Nucl Med Molecular Imaging.

[55] Zhao W, Zhong Z, Xie X, Yu Q, Liu J (2020). Relation between chest CT ndings and clinical conditions of coronavirus disease (COVID-19) pneumonia: a multicenter study. Am J Roentgenol.

[56] Zhou S, Wang Y, Zhu T, Xia L (2019). CT features of coronavirus disease 2019 (COVID-19) pneumonia in 62 patients in Wuhan, China. Am J Roentgenol.

[57] Xi Xu, Chengcheng Yu, Jing Qu, Zhang L, Jiang S, Huang D, Chen B, Zhang Z, Guan W, Ling Z, Jiang R, Tianli Hu, Ding Y, Lin L, Gan Q, Luo L, Tang X, Liu J (2020). Clinical and computed tomographic imaging features of novel coronavirus pneumonia caused by SARS-CoV-2. J Infect.

[58] Li Y, Xia L (2019). Coronavirus disease 2019 (COVID-19): role of chest CT in diagnosis and management. Am J Roentgenol.

[59] Yang W, Cao Q, Qin L, Wang X, Cheng Z, Pan A, Dai J, Sun Q, Zhao F, Qu J, Yan F (2020). Clinical characteristics and imaging manifestations of the 2019 novel coronavirus disease (COVID-19): a multi-center study in Wenzhou city, Zhejiang. China J Infect.

[60] Ai T, Yang Z, Hou H, Zhan C, Chen C, Lv W, Tao Q, Sun Z, Xia L (2020). Correlation of chest CT and RT-PCR testing for coronavirus disease 2019 (COVID-19) in China: a report of 1014 cases. Radiology.

[61] Li K, Wu J, Wu F, Guo D, Chen L, Fang Z, Li C (2020). The Clinical and Chest CT Features Associated With Severe and Critical COVID-19 Pneumonia. Invest Radiol.

[62] Xiong Y, Sun D, Liu Y, Fan Y, Zhao L, Li X, Zhu W (2020). Clinical and high-resolution CT features of the COVID-19 infection: comparison of the initial and follow-up changes. Invest Radiol.

[63] Bai HX, Hsieh B, Xiong Z, Halsey K, Whae Choi Ji, Tran TML, Pan I, Shi L-B, Wang D-C, Mei Ji, Jiang X-L, Zeng Q-H, Egglin TK, Ping-Feng Hu, Agarwal S, Xie F-F, Li S, Healey T, Atalay MK, Liao W-H (2020). Performance of radiologists in dierentiating COVID-19 from viral pneumonia on chest CT. Radiology.

[64] Cheng Z, Lu Y, Cao Q, Qin L, Pan Z, Yan F, Yang W (2020). Clinical Features and Chest CT Manifestations of Coronavirus Disease 2019 (COVID-19) in a Single-Center Study in Shanghai, China. Am J Roentgenol.

[65] Shi H, Han X, Jiang N, Cao Y, Alwalid O, Jin Gu, Fan Y, Zheng C (2020). Radiological findings from 81 patients with COVID-19 pneumonia in Wuhan, China: a descriptive study. Lancet Infectious Diseases.

[66] Wang J, Xu Z, Wang J, Feng R, An Y, Ao W, Gao Y, Wang X, Xie Z (2020). CT characteristics of patients infected with 2019 novel coronavirus: association with clinical type. Clin Radiol.

[67] Fan N, Fan W, Li Z, Shi M, Liang Y (2020). Imaging characteristics of initial chest computed tomography and clinical manifestations of patients with COVID-19 pneumonia. Jpn J Radiol.

[68] Qian L, Yu J, Shi H (2020). Severe acute respiratory disease in a huanan seafood market worker: images of an early casualty. Radiol Cardiothorac Imaging..

[69] Shi H, Han X, Zheng C (2020). Evolution of CT manifestations in a patient Recovered from 2019 novel coronavirus (2019-nCoV) pneumonia in Wuhan, China. Radiology.

[70] Lei J, Li J, Li X, Qi X (2020). CT imaging of the 2019 novel coronavirus (2019-nCoV) pneumonia. Radiology.

[71] Xu X, Yu C, Zhang L, Luo L, Liu J (2020). Imaging features of 2019 novel coronavirus pneumonia. Eur J Nucl Med Mol Imaging.

[72] Kong W, Agarwal PP (2020). Chest imaging appearance of COVID-19 infection. Radiol Cardioth Imag..

[73] Colombi D, Bodini FC, Petrini M, Maffi G, Morelli N, Milanese G, Silva M, Sverzellati N, Michieletti E (2020). Well-aerated Lung on Admitting Chest CT to Predict Adverse Outcome in COVID-19 Pneumonia. Radiology.

[74] Grillet F, Behr J, Calame P, Aubry S, Delabrousse E (2020). Acute pulmonary embolism associated with COVID-19 pneumonia detected by pulmonary CT angiography. Radiology.

[75] Oudkerk M, Büller HR, Kuijpers D, van Es N, Oudkerk SF, McLoud T, Gommers D, van Dissel J, Ten Cate H, van Beek EJ (2020). Diagnosis, prevention, and treatment of thromboembolic complications in COVID-19: report of the National Institute for Public Health of the Netherlands. Radiology.

[76] Léonard-Lorant I, Delabranche X, Séverac F, Helms J, Pauzet C, Collange O, Schneider F, Labani A, Bilbault P, Molière S, Leyendecker P, Roy C, Ohana M (2020). Acute pulmonary embolism in patients with COVID-19 at CT angiography and relationship to d-dimer levels. Radiology.

[77] Zhu T, Wang Y, Zhou S, Zhang N, Xia L (2020). A comparative study of chest computed tomography features in young and older adults with corona virus disease (COVID-19). J Thorac Imaging.

[78] Hansell DM, Bankier AA, MacMahon H, McLoud TC, Müller NL, Remy J (2008). Fleischner Society: glossary of terms for thoracic imaging. Radiology.

[79] Simpson S, Kay FU, Abbara S, Bhalla S, Chung JH, Chung M, Henry TS, Kanne JP, Kligerman S, Ko JP, Litt H (2020). Radiological society of north America Expert Consensus Statement on reporting chest CT findings related to COVID-19. Endorsed by the Society of Thoracic Radiology, the American College of Radiology, and RSNA. Radiol Cardiothorac Imaging.

[80] Salehi S, Abedi A, Balakrishnan S, Gholamrezanezhad A (2020). Coronavirus disease 2019 (COVID-19) imaging reporting and data system (COVID-RADS) and common lexicon: a proposal based on the imaging data of 37 studies. Eur Radiol.

[81] Prokop M, Van Everdingen W, van Rees VT, van Quarles Ufford H, Stöger L, Beenen L, Geurts B, Gietema H, Krdzalic J, Schaefer-Prokop C, Van Ginneken B, COVID-19 Standardized Reporting Working Group of the Dutch Radiological Society (2020). CO-RADS: a categorical CT assessment scheme for patients suspected of having COVID-19-definition and evaluation. Radiology.

[82] Li K, Fang Y, Li W, Pan C, Qin P, Zhong Y, Liu X, Huang M, Liao Y, Li S (2020). CT image visual quantitative evaluation and clinical classification of coronavirus disease (COVID-19). Eur Radiol.

[83] Fatima S, Ratnani I, Husain M, Surani S (2020). Radiological findings in patients with COVID-19. Cureus.

[84] Revel MP, Parkar AP, Prosch H, Silva M, Sverzellati N, Gleeson F, Brady A, European Society of Radiology (ESR) and the European Society of Thoracic Imaging (ESTI) (2020). COVID-19 patients and the radiology department—advice from the European Society of Radiology (ESR) and the European Society of Thoracic Imaging (ESTI). Eur Radiol..

[85] Wong HY, Lam HY, Fong AH, Leung ST, Chin TW, Lo CS, Lui MM, Lee JC, Chiu KW, Chung TW, Lee EY (2019). Frequency and distribution of chest radiographic findings in COVID-19 positive patients. Radiology.

[86] Li B, Li X, Wang Y, Han Y, Wang Y, Wang C, Zhang G, Jin J, Jia H, Fan F, Ma W, Liu H, Zhou Y (2020). Diagnostic value and key features of computed tomography in Coronavirus Disease 2019. Emerg Microbes Infect.

[87] Chinese Medical Association Radiology Branch Radiological diagnosis of new coronavirus pneumonia: expert recommendations from the Chinese Medical Association Radiology Branch (first edition). Chin J Radiol. (2020) 54:E001

[88] Phan LT, Nguyen TV, Luong QC, Nguyen TV, Nguyen HT, Le HQ, Nguyen TT, Cao TM, Pham QD (2020). Importation and human-to-human transmission of a novel coronavirus in Vietnam. N Engl J Med..

[89] Chen N, Zhou M, Dong X, Jieming Qu, Gong F, Han Y, Qiu Y, Wang J, Liu Y, Wei Y, Xia J, Ting Yu, Zhang X, Zhang Li (2020). Epidemiological and clinical characteristics of 99 cases of 2019 novel coronavirus pneumonia in Wuhan, China: a descriptive study. Lancet.

[90] Yoon SH, Lee KH, Kim JY, Lee YK, Ko H, Kim KH, Park CM, Kim YH (2020). Chest radiographic and CT findings of the 2019 novel coronavirus disease (COVID-19): analysis of nine patients treated in Korea. Korean J Radiol.

[91] Woznitza N, Nair A, Hare SS (2020). COVID-19: a case series to support radiographer preliminary clinical evaluation. Radiography.

[92] Rodriguez-Morales AJ, Cardona-Ospina JA, Gutiérrez-Ocampo E, Villamizar-Peña R, Holguin-Rivera Y, Escalera-Antezana JP, Alvarado-Arnez LE, Bonilla-Aldana DK, Franco-Paredes C, Henao-Martinez AF, Paniz-Mondolfi A (2020). Clinical, laboratory and imaging features of COVID-19: a systematic review and meta-analysis. Trav Med Infect Dis.

[93] Pakray A, Walker D, Figacz A, Kilanowski S, Rhodes C, Doshi S, Coffey M (2020). Imaging evaluation of COVID-19 in the emergency department. Emerg Radiol.

[94] Bandirali M, Sconfienza LM, Serra R, Brembillall R, Albano D, Pregliasco E (2020). Chest radiograph findings in asymptomatic and minimally symptomatic quarantined patients in Codogno, Italy during COVID-19 pandemic. Radiology.

[95] Filatov A, Sharma P, Hindi F, Espinosa PS (2020). Neurological complications of coronavirus disease (COVID-19): encephalopathy. Cureus.

[96] Poyiadji N, Shahin G, Noujaim D, Stone M, Patel S, Griffith B (2020). COVID-19- associated acute necrotizing hemorrhagic encephalopathy: CT and MRI features. Radiology.

[97] Manna S, Wruble J, Maron SZ, Toussie D, Voutsinas N, Finkelstein M, Cedillo MA, Diamond J, Eber C, Jacobi A, Chung M (2020). COVID-19: a multimodality review of radiologic techniques, clinical utility, and imaging features. Radiol Cardiothorac Imaging.

[98] Jones HA, Marino PS, Shakur BH, Morrell NW (2003). In vivo assessment of lung inflammatory cell activity in patients with COPD and asthma. Eur. Respir..

[99] Polverari G, Arena V, Ceci F, Pelosi E, Ianniello A, Poli E, Sandri A, Penna D (2020). 18F-FDG uptake in Asymptomatic SARS-CoV-2 (COVID-19) patient, referred to PET/CT for Non-Small Cells Lung Cancer restaging. J Thorac Oncol.

[100] Zou S, Zhu X (2020). FDG PET/CT of COVID-19. Radiology.

